# Development of a Novel Mobile Health App to Empower Young People With Type 1 Diabetes to Exercise Safely: Co-Design Approach

**DOI:** 10.2196/51491

**Published:** 2024-07-30

**Authors:** Vinutha B Shetty, Leanne Fried, Heather C Roby, Wayne H K Soon, Rebecca Nguyen, Arthur Ong, Mohinder Jaimangal, Jacinta Francis, Nirubasini Paramalingam, Donna Cross, Elizabeth Davis

**Affiliations:** 1 Department of Endocrinology and Diabetes, Perth Children's Hospital Telethon Kids Institute, University of Western Australia Division of Paediatrics, Medical School Perth City Australia; 2 Telethon Kids Institute University of Western Australia Perth City Australia; 3 Curve Tomorrow Perth Australia

**Keywords:** Mobile health application, Exercise, fitness, physical activity, design, co-design, focus group, focus groups, acT1ve, Type 1 diabetes, Young people, Blood glucose level, diabetic, diabetes, young, youth, type 1, prototype, develop, development, mHealth, mobile health, app, apps, applications, applications, user-centered design, mobile phone

## Abstract

**Background:**

Blood glucose management around exercise is challenging for youth with type 1 diabetes (T1D). Previous research has indicated interventions including decision-support aids to better support youth to effectively contextualize blood glucose results and take appropriate action to optimize glucose levels during and after exercise. Mobile health (mHealth) apps help deliver health behavior interventions to youth with T1D, given the use of technology for glucose monitoring, insulin dosing, and carbohydrate counting.

**Objective:**

We aimed to develop a novel prototype mHealth app to support exercise management among youth with T1D, detail the application of a co-design process and design thinking principles to inform app design and development, and identify app content and functionality that youth with T1D need to meet their physical activity goals.

**Methods:**

A co-design approach with a user-centered design thinking framework was used to develop a prototype mHealth app “acT1ve” during the 18-month design process (March 2018 to September 2019). To better understand and respond to the challenges among youth with diabetes when physically active, 10 focus groups were conducted with youth aged 13-25 years with T1D and parents of youth with T1D. Thereafter, we conducted participatory design workshops with youth to identify key app features that would support individual needs when physically active. These features were incorporated into a wireframe, which was critically reviewed by participants. A beta version of “acT1ve” was built in iOS and android operating systems, which underwent critical review by end users, clinicians, researchers, experts in exercise and T1D, and app designers.

**Results:**

Sixty youth with T1D, 14 parents, 6 researchers, and 10 clinicians were engaged in the development of “acT1ve.” acT1ve included key features identified by youth, which would support their individual needs when physically active. It provided advice on carbohydrates and insulin during exercise, information on hypoglycemia treatment, pre- and postexercise advice, and an educational food guide regarding exercise management. “acT1ve” contained an exercise advisor algorithm comprising 240 pathways developed by experts in diabetes and exercise research. Based on participant input during exercise, acT1ve provided personalized insulin and carbohydrate advice for exercise lasting up to 60 minutes. It also contains other features including an activity log, which displays a complete record of the end users’ activities and associated exercise advice provided by the app’s algorithm for later reference, and regular reminder notifications for end users to check or monitor their glucose levels.

**Conclusions:**

The co-design approach and the practical application of the user-centered design thinking framework were successfully applied in developing “acT1ve.” The design thinking processes allowed youth with T1D to identify app features that would support them to be physically active, and particularly enabled the delivery of individualized advice. Furthermore, app development has been described in detail to help guide others embarking on a similar project.

**Trial Registration:**

Australian New Zealand Clinical Trials Registry ACTRN12619001414101; https://tinyurl.com/mu9jvn2d

## Introduction

### Background

Physical activity has many physical and psychological benefits for young people living with type 1 Diabetes (T1D) [1]. In addition to the cardiovascular benefits, weight loss, and physical conditioning benefits, physical activity is associated with positive mental well-being, improved self-esteem, and quality of life [2-4]. However, the challenges experienced by young people living with T1D like fear of hypoglycemia can prevent them from being physically active [5-7]. Maintaining stable blood glucose levels (BGLs) before, during, and after exercise is affected by many factors and may not be predictable on repeated exercise occasions [8]. In addition, young people with T1D show significant decline in treatment adherence as they move through adolescence [9].

Several interventions have been developed to help young people with T1D effectively manage their physical activity [10]. However, few randomized controlled trials have explored the efficacy of these interventions [11]. Furthermore, most T1D interventions are educational, despite evidence that educational components alone, while necessary, are not sufficient in supporting adolescents when they are physically active [12]. Adding behavioral elements to an intervention, such as monitoring, goal setting, and linking medication taking with established routines, may enhance outcomes [12]. The use of mobile health (mHealth) technologies has become more common practice for diabetes self-management in middle- and high-income countries [13,14] enabling small to moderate improvements in glycemic stability, life satisfaction, and concern about diabetes and mental health [15-19].

### Apps and T1D

Apps to manage diabetes have become widespread and diverse in their functionality and purpose [18]. The recent consensus report from the joint European Association for the Study of Diabetes and the American Diabetes Association Diabetes Technology Working Group highlights the potential value of digital apps for diabetes self-management [20]. An exercise advisor app for those living with TID has recently been designed [21], and there are an increasing number of apps that give guidance to people living with T1D during exercise [22].

Apps that target specific age groups are needed to help in the management of physical activity and T1D [23]. Adolescents living with T1D have specific health and developmental needs related to the many physical, psychological, and neurodevelopmental changes they are undergoing. They are not only becoming focused on peer relationships but are also developing a need for autonomy and have unique challenges adhering to T1D management regimes [24]. Therefore, apps for this age group need to be specifically tailored.

*Bant* (Centre for Global eHealth Innovation from University Health Network Toronto), an app designed for young people with T1D collects information on physical activity with no specific design aspect for exercise [12]. While the app T1D Exercise focuses on exercise management strategies, it is only designed for adults [22]. Previous formative research by our team provided anecdotal evidence that young people living with T1D would use an app, if suited to their needs, to help them manage their BGLs before, during, and after physical activity [25]. Until recently, there were no apps that specifically support diabetes self-management and provide individualized information around exercise in young people living with T1D. Diactive-1 App has been recently developed by a team of researchers from University of Turin, Italy. This app which is not commercially available is being tested to explore the effect of this app intervention on insulin dose requirements in children and adolescents with T1D [26].

### User-Centered Design

User-centered design (UCD) of mHealth apps recognizes that all innovation starts with a deep and comprehensive understanding of the end user, such as their current behaviors, motivations, their feelings and emotions, intentions, what technology they depend on, who is important to them, and their self-image [27]. In UCD, the needs, abilities, and desires of the end user drive the design at each stage of the process [28]. Design thinking is a UCD process that encourages iterative exploration of solutions, continual refinement of the problem, and increased understanding of user needs [29]. This approach in app development may ensure the necessary relevance and robustness of the app. This is important given that with high drop-out rates of mHealth app use [30], more effort is needed to incorporate the needs, experiences, and expectations of end users in app development [31,32]. A recent integrative review questioned whether mHealth was indeed being used to address the target audiences’ needs [33].

UCD and co-design can be used together to maximize app relevance. A co-design approach to designing and developing mHealth apps allows people who are the end users of the app, providers who are the health care professionals, and researchers to contribute their collaborative insights [34]. Co-design (also referred to as participatory design) is a key research methodology that enables the perspectives and preferences of the target end user population to influence subsequent development of the mHealth app. Ideally, it involves a process of shared decision-making, which is characterized by health care professionals and end users collaborating to make decisions about the mHealth app, with a balanced focus on both hard-clinical evidence as well as the end user’s priorities and values [35]. This suggests the necessity of engaging both end users and health care professionals in co-design to design and develop the technology they will use together [36]. Although the number of available mHealth apps is increasing rapidly, there is a need to create a rigorous design thinking process for their development [29] and guidance that ensures the sustainability of the apps [37,38].

Emerging apps do provide exercise support. However, none of the currently available apps exclusively provide tailored advice on exercise for young people with T1D. Hence, the aim of the study was to apply a co-design process and design thinking principles to build a novel app for young people living with T1D that delivers individualized diabetes management advice to support physical activity. The objectives of the study were to (1) detail the application of a co-design process and design thinking principles to inform the design and development of the app and (2) identify the content and functionality that young people living with T1D need in the app to meet their physical activity goals.

## Methods

### Recruitment

Adolescents and young adults aged 13-25 years with a diagnosis of T1D for more than 6 months and competent in speaking English were eligible to participate. Multiple recruitment strategies were used to provide broad representation. Eligible participants were identified through the Western Australian Children’s Diabetes Database, a state-wide, population-based, longitudinal diabetes registry. Participants were provided with participation information sheets via email, or when approached by researchers at diabetes clinics at Perth Children’s Hospital. The studies were also advertised on local diabetes organization websites and social media. Recruitment of focus group participants continued until data saturation was attained. In the subsequent phases, recruitment concluded when all recruitment methods were exhausted. 

### Ethical Considerations

Participants provided consent in accordance with the Child and Adolescent Health Service Human Research Ethics Committee, registered with the National Health and Medical Research Council’s Australian Health Ethics Committee. Parental consents were also obtained for participants younger than 18 years. Ethical approval of this project (RGS0000000743) from the Child and Adolescent Health Service Human Research Ethics Committee was valid from January 4, 2018, to January 4, 2021, subject to compliance with conditions of Ethics Approval for a Research Project.

### Study Design

#### Overview

**Figure 1:**
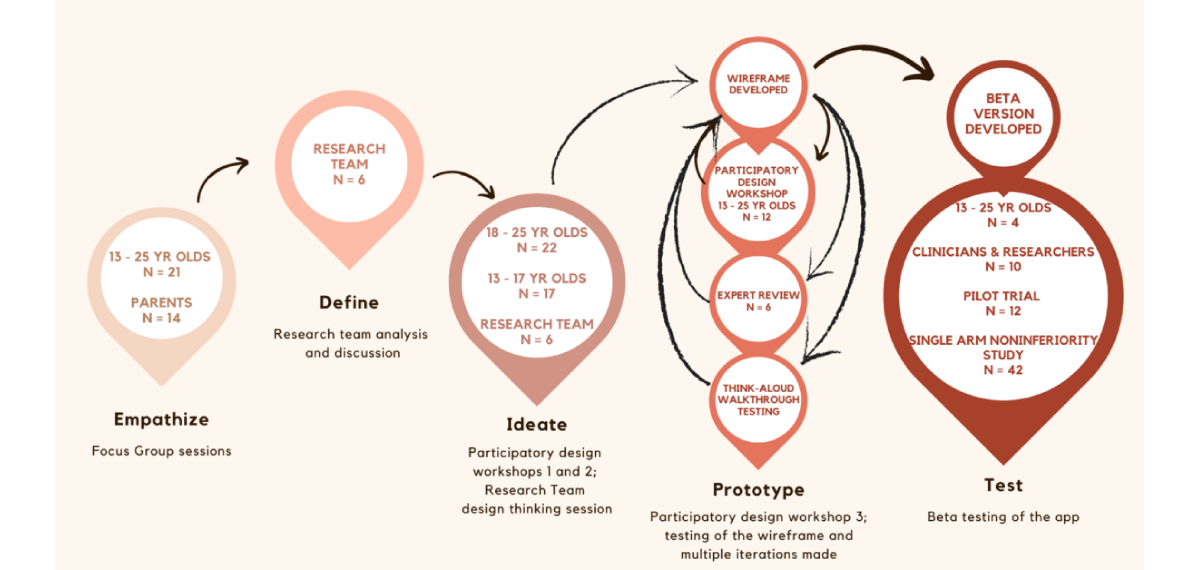
The 5-stage design thinking process.

A co-design approach and design thinking framework (Figure 1) were used, supported by UCD methodology to understand the requirements of the end users and to iteratively design the app. The user-centered mixed methods included focus groups, participatory design workshops, and acceptability and usability evaluation methods. These methods were iteratively applied during the 18-month design process from March 2018 to September 2019.

A UCD framework based on the Stanford Design Thinking 5-stage (Empathize, Define, Ideate, Prototype, and Test) methodology [39] was used to develop the app (Figure 1).

#### Empathize

The Empathize stage in design thinking is to provide greater insight into the identified problem and gain a deep understanding of the end user's needs, emotions, and experiences.

In the Empathize stage, focus groups were conducted with young people with T1D with ongoing input from the research team, to better understand end users’ perspectives of diabetes management around exercise, especially the challenges they faced while being physically active. Participants were asked questions such as: *What are the main difficulties you have when physically active? What works for you when you are physically active?* Thematic analysis was undertaken to synthesize the feedback from the focus group.

#### Define

The purpose of the Define stage is to use the findings from the Empathize stage to more precisely define the problem statement to guide the subsequent stages of the design process. In the Define stage, a series of structured and unstructured discussions were undertaken by the research team using the thematic analysis of data collected to (1) define the problem statement, (2) confirm details of the target audience, (3) outline the needs of the target audience, and (4) determine how these needs were to be met.

#### Ideate

In the Ideate phase, generation of a wide range of creative ideas and concepts is the main goal. Judgement and evaluation are deferred to encourage the target audience to develop a broad range of possible solutions. In the ideate stage, 2 participatory design workshops were conducted to identify features of an app that support the target group to be physically active, and to translate existing clinical guidelines into an exercise advisor algorithm providing targeted and individualized recommendations in the app. Participants were organized into mixed-sex groups, each with a cofacilitator.

A total of 22 young adults aged 18-25 years and 17 adolescents aged 13-17 years with T1D participated in the first and second participatory design workshop, respectively*.* The research team facilitated and recorded the process. The questions and activities were tailored to the age groups and included individual responses and affinity paired or small-group discussions. Data were collected primarily using a nominal group process whereby participants generated and recorded their ideas individually, and then discussed and voted on these ideas as a group.

In the first workshop, participants were asked questions (Multimedia Appendix 1) about what technology motivates them during physical activity, the challenges faced during physical activity, specific roadblocks they experienced trying to solve the challenges, the source of the help or information they received to address the challenges, and what they would want from an exercise app if they were to design one. They were then presented with a physical activity “tree” (or commonly called a “user journey flow” in design thinking methodology), that outlines the steps, interactions, and touch points an end user goes through while engaging with a product, service, or experience, providing a holistic understanding of their overall experience. Participants were instructed to map their own physical activity routines and diabetes management plans and share it with a partner, then with the group. Participants gave feedback on the flow and identified priority areas where they needed help and where an app could support them. Activities included brainstorming and designing paper prototypes for the app.

The second workshop followed the same format as the first, with different questions (Multimedia Appendix 1) and tasks. A “scenario approach” was used where participants were instructed to draw a flowchart of their last physical activity, describe the steps taken to manage their glucose levels, and identify and address any roadblocks encountered. Features of an app that might help them to be physically active, manage T1D, and solve roadblocks were recorded individually and then shared with the group. At the end of the group discussions, comments were presented to the whole workshop.

The Ideate stage also included a design thinking session with a digital health officer and a subgroup of the research team (n=6) using a brainstorming process that considered requirements and constraints to identify ways of developing the app. In addition to discussing the structure of the exercise advisory algorithm to be incorporated in the app, this process considered the major themes that arose from the participatory design workshops, considering how young people would use technology to make decisions about diabetes management around exercise and to determine features that could be included.

#### Prototype

The Prototype phase is an experimental phase, designed to identify the best possible solutions to address the problems. The aim was to use the findings from the Ideate stage to rapidly build and test low-fidelity prototypes to explore potential solutions and gather feedback for further iteration. In the Prototype stage, a wireframe prototype (simple mock-up version) and beta versions of the app (“beta” means the app is under development) were developed based on the input from the participatory design workshops. This phase aligns with one of the principles of design thinking—*The tangibility rule*: Making ideas tangible in the form of prototypes to enable designers to communicate more effectively with the target audience [40]. UCD methods commonly used in contemporary design were used. These entailed the iterative involvement of the end user in the design process by eliciting formal feedback on reference and prototype versions of the app and formative usability testing of the system. Participants from the initial 2 workshops were invited back to participate in a third participatory design workshop (n=12) to provide this feedback.

#### Test

The prototype stage was followed by developing a fully functional app in the Test stage that could be downloaded, tested, and iterations made based on feedback. The beta version of the app was developed and tested in the final fourth workshop by 10 clinical and research staff and 4 young people with T1D who had attended the previous 3 workshops. The app was modified based on the feedback from beta testing. The final app was then tested in a pilot trial.

The app was piloted in a free-living setting to assess its acceptability, functionality, and gather feedback on the user experience. The trial participants were 10 adolescents and young adults (aged 12-25 years) living with T1D who had not attended any of the participatory design workshops. They tested the app in a free-living setting and were asked to use the app to guide their exercise management for 6 weeks. At the end of 6 weeks, all participants completed both a semistructured interview and the user Mobile Application Rating Scale (uMARS) [41]. All semistructured interviews were transcribed. Thematic analysis was conducted whereby interview transcripts were independently analyzed by 2 researchers to uncover relevant themes. The uMARS was scored for the 6 quality subscales (engagement, functionality, aesthetics, information, perceived impact, and subjective quality). All scores have a maximal possible value of 5, and were presented as medians, IQRs, and ranges [42].

An important aspect of this study design was the ongoing engagement of young people as co-designers in the design, implementation, and evaluation of the wireframe and then fully functional app. The process moved from generating insights about end users to idea generation and testing [43].

## Results

### Empathize: End User Focus Groups

To identify the challenges faced by young people living with T1D when physically active, 6 focus groups were conducted with a total of 14 adolescents and 7 young adults with T1D. Four focus groups were also conducted with 14 parents of the adolescents. Thematic analysis of focus group data suggested many challenges experienced by young people living with T1D when physically active and the results are reported elsewhere [25]. Briefly, these included, managing food and equipment, managing glucose levels with changing environment conditions, and psychosocial challenges. Parents also experienced their own challenges when their adolescents were physically active, including supporting them to be autonomous. These challenges were revisited in later phases of the design process to inform the presentation and support for the physical activity guidelines.

### Define: Research Team Discussions

Clinicians and researchers were involved in this stage (n=6). Analysis of the discussion data were used to define the following:

1. Problem statement: young people living with T1D are challenged by the unpredictability of their BGLs before, during, and after physical activity. This unpredictability is caused by various physical, environmental, and psychosocial factors. To manage this unpredictability, young people need guidelines and support to implement these guidelines. The research problem involves finding the best means of presenting young people with personalized guidance on insulin dosing and carbohydrate intake strategies for facilitating diabetes management around exercise.
2. Target audience: young people living with T1D aged between 13 and 25 years who are, or who would like to be, physically active.
3. Needs of the target audience: to maintain stable BGLs before, during, and after physical activity by building their understanding of their blood glucose response patterns to varying types, duration, times, and intensities of physical activity.
4. How their needs were to be met: deciding the best way to meet the identified needs was through the development of an app to deliver individualized advice on insulin dosing and carbohydrate intake during and after physical activity.

### Ideate: Participatory Design Workshops

Workshop duration was approximately 90 minutes for each. In total, 22 young adults aged 18-25 years (mean age 20.4 years) with a diagnosis of T1D participated in the first participatory design workshop, and 17 adolescents aged 13-17 years (mean age 14.5 years) with a diagnosis of T1D participated in the second workshop.

The research team reviewed the individual and group responses as well as the facilitator notes from the participatory design workshops and developed a list of desirable app features that participants indicated they needed to overcome the challenges experienced when physically active (Table 1). The team then selected the features rated the highest by the participants to incorporate into the first version of the app.

**Table 1:** Desirable app features from Ideation phase.

Desirable app features	Examples
Support music	Can receive advice for different activities Can search for answers to questions Listen to music while tracking blood glucose levels
Diet tracker	Counts carbs and suggests foods
Blood glucose levels	Measures effects of different types of exercise on blood glucose and tracks patterns
Communication	Communicates with CGM^a^ and Fitbit

^a^CGM: Continuous glucose monitor.

In the software development process, an exercise advisory algorithm with 240 pathways was created based on the decision tree from the published international consensus guidelines [44-46]. The algorithm expanded from a skeletal exercise activity mapping tree to separate decision trees for 4 different types of exercise: mild, moderate, mixed-intermittent intensity, and resistance exercise of up to 60 minutes in duration. Personalized insulin dose and carbohydrate advice is generated by the decision tree algorithm based on the end user’s weight, type, intensity and duration of physical activity, the duration since the last insulin bolus, and the participant’s glucose levels at the start of activity. In addition, more information on hypoglycemia treatment, pre- and postexercise insulin and carbohydrate advice, and an educational food guide which highlights the importance of low and high–glycemic index foods in the context of exercise was developed to be incorporated into the app.

### Prototype

#### Developing the Wireframe

With the knowledge generated from the workshops and input from the research team, the digital health officer developed a “wireframe” of the app. A wireframe is a simplified visual representation or blueprint that outlines the basic structure, layout, and content placement of a web page or application, focusing on functionality and information hierarchy rather than visual design. End users reviewed a set of images displaying the functional elements of the app on their smartphones. The end users included participants from the initial 2 workshops who were invited back to participate in a third participatory design workshop (n=12) and researchers (n=6) through individual and group feedback processes. The wireframe was refined after each group’s review.

**Figure 2:**
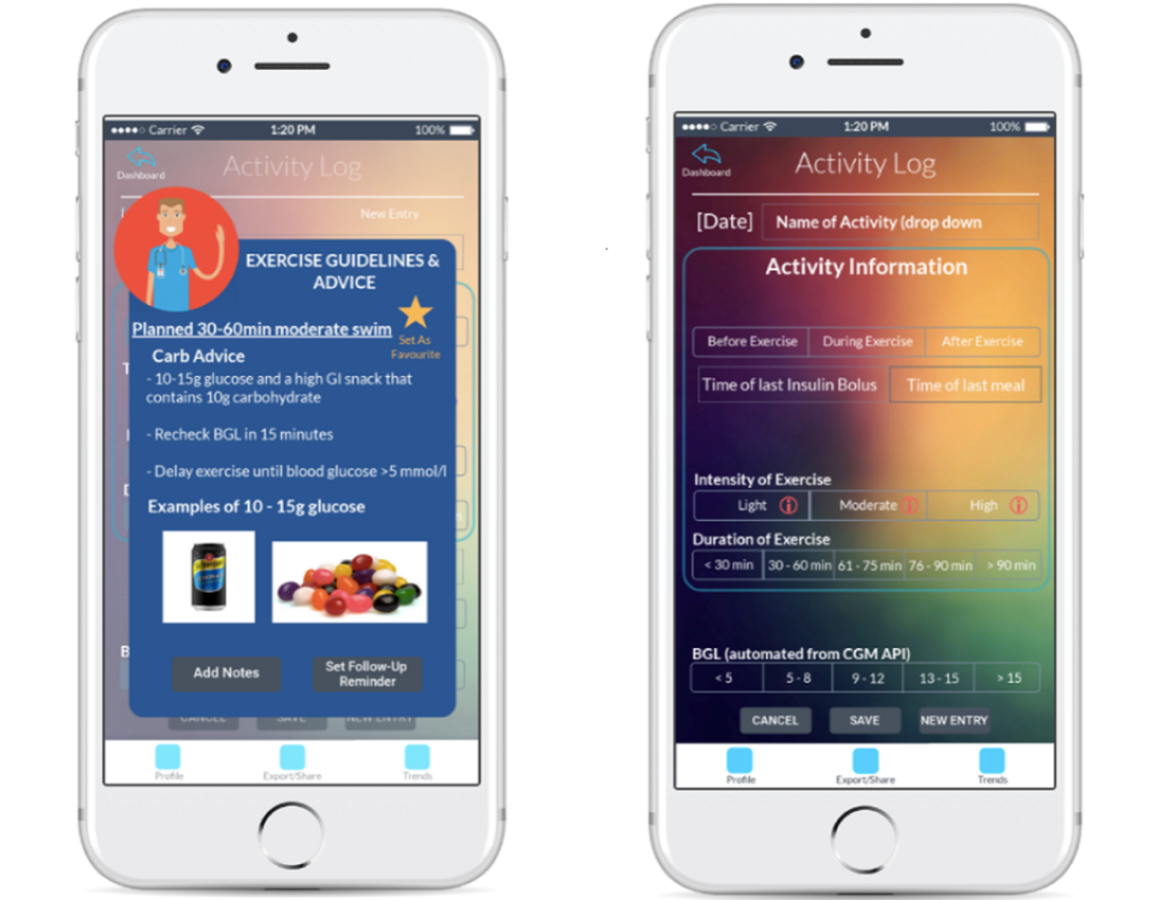
Wireframes of the app showing exercise advice and Activity log.

The objective of this third workshop was for the participants to assess the wireframe tool on an iOS device and to determine whether it was appropriate for further development and testing as a smartphone app. A total of 12 adolescents and young adults with T1D aged 13-25 years (8 females), attended a 2-hour workshop, and were asked to test the wireframe on an iOS device (iPhone or iPad). Participants were given time to explore the wireframe individually and then discussed their impressions in small nominal groups (mean group size=4). Figure 2 shows an example of a feature presented in the wireframe. The screenshots show an example of the exercise advice provided at the start of exercise and the activity log feature where all the information of the activity undertaken by the end user is recorded and available for future reference (Figure 2).

Participants were then asked to respond to more targeted questions about each of the functions integrated into the wireframe including the Activity Log, Quick Advice, Music, What Worked, Profile, Notifications, and Quick View, provide comments on what they liked or did not like and what functions could be improved. Individual, group, and facilitator notes were collated and analyzed to inform wireframe version 2.0 of the prototype. These findings are shown in Table 2. Research team members, pediatric endocrinologists, and a digital health officer independently reviewed wireframe version 2.0 over a week and then met to discuss the flow, content, and aesthetics of the app. A list of suggestions to improve the app was generated and aligned with the aim of the project.

**Table 2:** Feedback from the third participatory design workshop.

Feature	Liked	Did not like	Suggestions for improvement
Activity log	Clearly set up and easy to follow	Do not understand the facial expressions of exercise intensity	Could include a regular activity profile—a typical week
Music	Being able to set the duration of the music	The general aesthetics	Link to your own music library
Quick advice	How you click on previous activity and get advice	It took a bit of time to get the advice	A different look including different icons
Notifications	The inspirational quote	The design of the buttons	Include a change in quotes and pictures
Profile	The ability to set goals		Include time frame to achieve goals
What worked	Clearly showed important information about the activity	7 days and 30 days could be changed to a week and a month	Should be able to record BGLs^a^ and information about what worked in relation to advice given
Quick view	Very helpful	Information too squashed	Needs more pages

^a^BGLs: blood glucose levels.

#### App Development

Following the involvement of the researchers and end users in the iterative design process by eliciting formal feedback on reference and the wireframe of the app, the app scope was developed. Feedback collected through testing the wireframe was synthesized and used to inform the development of the app. Insights gained from the end users and researcher feedback were organized into functional components, informing requirements of the app’s design in a product roadmap. The research team met with several app development companies to ensure the scope and functional requirements of the app were fully understood during the tender process. Following the tender process, the digital health company “Curve Tomorrow” developed the beta version of the app. The software development process involved the app developers actively engaging with the endocrinologist and researchers weekly for 4 months to understand and develop the different elements of the complex exercise advisory algorithm in a functional way in the app and to ensure they understood the needs of the end users. The process was an ongoing 2-way communication until the app was fully functional.

The end users and the research team provided a list of desired features and functionality for development, but when these features were investigated in more detail by a business analyst, it was discovered that the feature requests were more detailed, complex, and likely to require additional funding to develop. The features were prioritized by the team based on the highest end user engagement and needs analysis, denoted as must-have requirements for the pilot trial such as additional information for each exercise, against nice-to-have functionality such as adding decision-making support through artificial intelligence. Even during this refinement process, further investigation, and analysis to understand the complex functional requirements was needed. This iterative process required collaboration from end users, researchers, and the app development team. During this refinement process, the aim was to ensure the end users felt their needs were met while considering budgetary constraints. After confirmation, the nice-to-have features which were not viable for the pilot trial were reserved for future additions. Following the 4-month period of software development, a further process to prioritize the features based on cost or benefit was applied, a preliminary list of features was agreed to, and the process proceeded toward beta development.

### Test

#### Beta Testing of App

In the final workshop, the beta version of the app was tested by 4 participants from the initial 2 workshops and 10 clinical and research staff. All test participants were given the beta version of the app to explore and then respond to the “think-aloud” process, which involves observing and recording an individual's thought processes and verbalizations as they interact with the app or perform a task to provide insights into their cognitive processes and end user experience. When participants expressed emotion, the researcher would ask, for example, “Why is that frustrating you? What did you expect to happen?” or “What would you prefer instead?” or “How would you make it better?” All feedback and suggestions for improvement were collated into 1 spreadsheet and summarized.

The research team then presented the feedback suggested by the majority of the app testers to the app developer. Feedback presented was quite minor with adjustments such as content changes, amendments to questions in the profile set up, some visual amendments (buttons being larger), and renaming menu names. These modifications were made to the app to enable pilot-testing. Following feedback from end users, researchers, and clinicians, the app was named “acT1ve.”

#### Pilot Trial

In total, 10 individuals (8 females and 2 males) were enrolled in this study and had a mean age of 17.7 (SD 4.2) years, T1D duration of 7.2 (SD 4.8) years, and hemoglobin A_1c_ of 54 (5.5 mmol/mol) (7.1 ± 0.5%) and engaged in physical activity for 4.5 ± 2.9 hours per week. All 10 participants had acT1ve installed on an Apple iPhone. No participants stopped using the app before the end of the 6-week period.

The qualitative and quantitative analysis of this study provided an important insight into the perspectives of participants in relation to the functionality and usability of the app. The information provided by the app was found to be relevant, appropriate, and clear, with a simple and easy flow of presentation. Participants felt they received adequate information to guide their diabetes management and enable them to maintain stable BGLs during physical activity. This reduced their worry about their glucose levels and provided them with trusted information and confidence to be more physically active. The participants liked the design of the app and found it acceptable and useful. They indicated that they would continue to use it long term and recommend it to friends and other people with T1D. The suggestions provided by the participants for improvement of the app were: a help section on how to use the app for those who may need extra guidance, added information for longer duration of exercise of more than 60 minutes of activity, minimization, and simplification of some of the information, suggestions for data sharing and social interaction features, and improving aesthetics.

The uMARS scores for acT1ve were high (out of 5) for its total quality 4.3 (4.2-4.6), engagement 3.9 (3.6-4.2), functionality 4.8 (4.5-4.8), information 4.6 (4.5-4.8), aesthetics 4.3 (4.0-4.7), subjective quality 4.0 (3.8-4.2), and perceived impact 4.3 (3.6-4.5). The pilot results showed that the acT1ve app is functional and acceptable with high user satisfaction. Details of this pilot study are published in a separate paper [42].

acT1ve was amended following the feedback from the pilot study participants. The amended app has been tested for safety and efficacy in a larger clinical trial (single arm noninferiority study) with 42 adolescents and young adults (aged 12-25 years) living with T1D in free-living environment. Once safety is established, regulatory approvals will be obtained prior to introducing the app to regular clinical practice.

## Discussion

### Principal Findings

This paper describes the co-design approach and the practical application of the *5-stage design framework* in developing an app to support young people living with T1D to be physically active. The process enabled the development of a unique exercise app that provides individualized insulin and carbohydrate advice and builds a record of “what works” for various activities, potentially facilitating a greater level of end user confidence when physically active. It is intended that just prior to exercise, youth living with T1D would answer questions about the exercise they are about to complete, and their diabetes management at that point in time, and would be provided with personalized insulin dose and carbohydrate advice for exercise. As the relationship between exercise and T1D is very complex, it is expected that end users would engage with the app for each exercise session, especially when commencing a new form of exercise.

Young people with T1D identified desirable features of the app and provided suggestions to improve the individual advice given, including linking it to their own continuous glucose monitor (CGM) and Fitbit, and the recording of responses to advice to build a better understanding of the individualized way their body responds to physical activity. They also wanted the app to count carbohydrates and suggest foods to eat before, during, and after exercise and to provide a search function so users can ask a range of questions. Additionally, they identified the desire to personalize the appearance of the app where possible and to be able to listen to music while using the app.

### Project Contribution

This project and the acT1ve app are unique for 4 reasons: the app focuses solely on exercise management for young people with T1D, a “state of the art” design thinking process was used in the development of the app, individualized advice is provided to end users and a pilot testing phase was included. In a recent study of m-health apps for people with a chronic condition only a small number included a pilot testing phase [31]. There are apps on the market that involve young people at each stage of the participatory design process [47] such as the *Bant* app for T1D [12]. However, unlike our project, the effects of involvement on acceptability were not examined for this app.

The failure of health information technology interventions in the past has been largely due to poor design that did not meet the requirements of the users of the technology [28]. In this co-design project end-user’s needs drove the app development, including their input at each stage of the process. To strengthen the robustness of the app, each stage of this project was informed by the previous stage and design iterations were undertaken to refine the app to meet the needs of young people with T1D. The continual synthesis of end user’s feedback with health professional’s advice has enabled the development of a potentially usable and useful app, with high end user satisfaction, as confirmed in the pilot trial [42].

### The Design Process and Adolescents

The importance of the participatory design process is highly relevant for the adolescent T1D population. The blood glucose response to physical activity can be highly individualized for young people living with T1D [48], hence the need to involve a group of adolescents and young adults with T1D of different ages and gender in the process of developing the app. Involving adolescents with T1D in a participatory design process enhances their empowerment and addresses their need for autonomy. Feelings of empowerment and autonomy are associated with improved psychological outcomes for young people with T1D [49]. Novel approaches as used in the initial workshops, that is, a “scenario” approach—are also suited to young people who particularly like real-world and relevant activities [50].

### Limitations

Several cofacilitators were needed to conduct the participatory design workshops which contributed to different levels of participant engagement. At times, the participatory design workshops were dominated by more vocal participants, often the older participants, and it is possible that less vocal participants were not able to fully express their viewpoints. This is a well-documented limitation of focus groups that can be offset by experienced facilitators [51]. There was also, in one of the participatory design workshops, a discrepancy in the power relations between adults and young people, with a considerable number of adults present. Adult numbers need to be minimal in participatory design processes with young people to encourage a sense of equality among the participants [52].

Designing an application to display complicated information is challenging. The additional barrier of presenting digestible information for the younger audience on a mobile format required omissions to what could be displayed, such as necessary language to be used being unable to fit the character limits of mobile devices. Additional challenges involved in using mobile devices included limiting the number of devices to be tested on and compatibility issues with operating software updates. Due to limited budget and certain logistic issues, certain features which could increase user engagement (ie, nice-to-haves) like integration with real-time CGM, activity tracker, and data sharing options were deprioritized for future modifications.

### Conclusions

This paper describes a co-design approach using design thinking processes undertaken to develop an mHealth app to support young people with T1D when they are physically active. The design thinking processes allowed young people with T1D to identify app features that would support them to be physically active, and particularly that enabled the delivery of individualized advice. In addition, the process of app development has been described in detail to help provide guidance to others embarking on a similar project. Use of the design thinking process has the potential to create highly usable mHealth apps that can improve health behaviors in various populations of young people, especially those who need to self-manage chronic conditions such as diabetes while increasing the relevance of the content and empowering those involved in the process.

## Appendix

Multimedia Appendix 1 Questions from participatory design workshops.

## Abbreviations

BGL: Blood glucose levels
CGM: Continuous glucose monitor
mHealth: Mobile health
T1D: Type 1 diabetes
UCD: User-centered design
uMARS: User mobile applications rating scale
