# Supplementary material for: Development of a Novel Mobile Health App to Empower Young People With Type 1 Diabetes to Exercise Safely: Co-Design Approach
Source: JMIR Diabetes. 2024 Jul 30;9:e51491. doi: 10.2196/51491 (PMC11322682; doi:10.2196/51491)
Supplement: Multimedia Appendix 1 [file diabetes_v9i1e51491_app1.docx]

**Appendix 1**

**Questions from Participatory Design Workshop**

Workshop 1 (18-25year olds):

1. What technology motivates and supports you to be physically active?
2. Think about a situation when you last did physical activity. This could be at school during physical education, walking the dogs, going for a walk etc. a) Can you briefly describe the scenario? b) Were there any issues or challenges in relation to your diabetes? How did you feel? c) What specific roadblocks did you experience trying to solve the challenges? d) If you didn’t have challenges how did you avoid them?
3. Where do you go for help/get information when you have challenges when physically active?
4. What sort of help or information would you like when physically active?
5. If you could design an app to help you when physically active, what would you want it to do?

Workshop 2 (13-17year olds):

Draw a flow chart of your last physical activity & steps taken to manage T1D, & any roadblocks.Share with partner or group

On your flowchart, write about what you tried to do at the roadblock.

In groups: Look at the group’s roadblocks. If you could have anything, what would you need to help solve these?

What technology do you use when physically active?

What features of this are important to you?

What technology supports do you need/would like when physically active?

Where do you go to get information about physical activity? Social media accounts? Blogs?
